# First Case Report of Prader–Willi-Like Syndrome in Colombia

**DOI:** 10.3389/fgene.2018.00098

**Published:** 2018-03-21

**Authors:** Estephania Candelo, Max M. Feinstein, Diana Ramirez-Montaño, Juan F. Gomez, Harry Pachajoa

**Affiliations:** ^1^Health Sciences Faculty, Universidad Icesi, Cali, Colombia; ^2^Health School, Case Western Reserve University School of Medicine, Cleveland, OH, United States; ^3^Paediatric Neurology, Fundación Valle Del Lili, Cali, Colombia; ^4^Genetics Department, Fundacion Valle del Lili, Cali, Colombia

**Keywords:** Prader–Willi-like syndrome, Prader–Willi-like phenotype, Prader–Willi syndrome (PWS), 6q16.1–q21 deletion, pediatric obesity

## Abstract

**Background:** Prader–Willi-like syndrome (PWLS) is believed to be caused by a variety of disruptions in genetic pathways both inside and outside of the genetic region implicated in PWS. By definition, PWLS does not demonstrate mutations in the 15q11–q13 region itself. It is a rare disorder whose clinical hallmarks include hypotonia, obesity, short extremities, and delayed development. This syndrome has been described in patients with 1p, 2p, 3p, 6q, and 9q chromosome abnormalities and in cases with maternal uniparental disomy of chromosome 14 and fragile X syndrome.

**Case presentation:** In the present report, we describe a 9-year-old Colombian patient who demonstrated features of PWS and was ultimately diagnosed with PWLS after genetic analysis revealed a 14.97 Mb deletion of 6q16.1–q21.

**Conclusions:** This is the first reported case of PWLS in Colombia and represents one of the largest documented 6q21 deletions.

## Background

Prader Willi-Like Syndrome (PWLS) is a rare disorder that whose clinical hallmarks include hypotonia, obesity, short extremities, and delayed development (Cheon, 2016). As its name suggests, the clinical presentation of PWLS shares features with that of the genetic imprinting disorder Prader Willi Syndrome (PWS). Despite sharing phenotypic characteristics, these diseases should be distinguished by clinicians because different treatments may be warranted for each one. Therefore, clinicians are challenged to use clinical presentation as a basis for determining best diagnostic and treatment options.

PWS is a disorder whose etiology is a lack of expression of paternal genes on chromosome 15q11-q13 (Rocha and Paiva, 2014). The three main classes of chromosomal abnormalities that result in PWS include microdeletion (65–75% of patients), uniparental disomy (20–30% of patients), and imprinting defects (1–3% of patients; Cheon, 2016). Clinical manifestations of PWS occur in two phases. The first phase is a neonatal hypotonia that often resolves by 8–11 months of life. The second phase is characterized by hyperphagia leading to obesity, small dysmorphic facial such as almond-shaped eyes or strabismus, neuropsychomotor delay, short stature, small hands and feet, developmental delay, hypopigmentation, and delayed onset of puberty (Rocha and Paiva, 2014).

PWLS is thought to be caused by a variety of disruptions in genetic pathways both inside and outside of the genetic region implicated in PWS. By definition, PWLS does not demonstrate mutations in the 15q11-q13 region itself (Berends et al., 1999).

The clinical overlap between both syndromes makes it difficult to accurately diagnose, the correct diagnosis may help to an appropriate management, but also to provide conclusive genetic explanation and accurate genetic counseling. Molecular techniques such as fluorescence *in situ* hybridization (FISH) or chromosomal microarray (CMA) can provide an accurate diagnosis of a chromosomal deletion indicative of PWS. The specific molecular cause of PWS can be diagnosed with the PCR-based methylation test (PBMT) to amplify 15q11-q13 (Rocha and Paiva, 2014). By contrast, a diagnosis of PWLS is made in the presence of the PWS phenotype but the absence of PWS-defining mutations in the 15q11-q13 region.

In the present report, we describe a 9 year-old Colombian boy who demonstrated the findings of PWS and was ultimately diagnosed with PWLS after genetic analysis revealed a 14.97 Mb deletion from 6q16.1-q21. This is the first reported case of PWLS in Colombia, and represents one of the largest documented 6q21 deletions.

## Case presentation

The proband is the first child of healthy non-consanguineous Colombian parents born to 38th weeks. Pregnancy and vaginal delivery were uncomplicated with Apgar scores of 8 and 9 at 1 and 5 min, respectively. His birth weight was 3.1 kg (32nd centile) and height was 51 cm (48th centile). At birth, he was noted to have neonatal hypotony. During a follow-up visit at 3 months, psychomotor delay, strabismus and short stature were noted. He started exhibiting motor delay, achieving sitting at 12 months, crawling at 15 months and walking at 3 years. Later, his speech development was also noted to be compromised and currently he can say only one or two words. At 24 months, the patient underwent corrective surgery for strabismus. After that, at 32 months, a diagnosis of severe intellectual disability was made based on the Wechsler Intelligence Scale for Children (WISC). The results of this test showed verbal score of 46, average score of 46 and WISC full-scale IQ score of 40. The patient underwent a brain CT and metabolic testing, the results of both of which were within normal limits. Because of his short stature and signs of insulin resistance, the proband underwent a endocrinological evaluation at 9 years old, which included total serum cholesterol 248 md/dL (<170 mg/dL), glucose test pre-meal 7 mg/dL (80–130 mg/dL), triglycerides 157 mg/dL (<90 mg/dL), HDL 38.3 mg/dL (>45 mg/dL), LDL 178.3 mg/dL (<110 mg/dL), and TSH 3.38 mIU/mL (0.66–4.14 mIU/mL).

At age 7, G-banded karyotype analysis revealed 46,XY, del(6)(q27). At age 9, analysis of bone age revealed 5 years. Also at age 9, was performed a high resolution (750K) Affymetrix GeneChip 6.0 array, that revealed a deletion from 6q16.1-q21 that was 14, 96 Mb pairs (chr6: 92, 310, 231-107, 849, 959). The following genes were compromised in this deletion: *SIM1, GRIK2, POPDC3*, and *MCHR2*.

**Figure 1:**
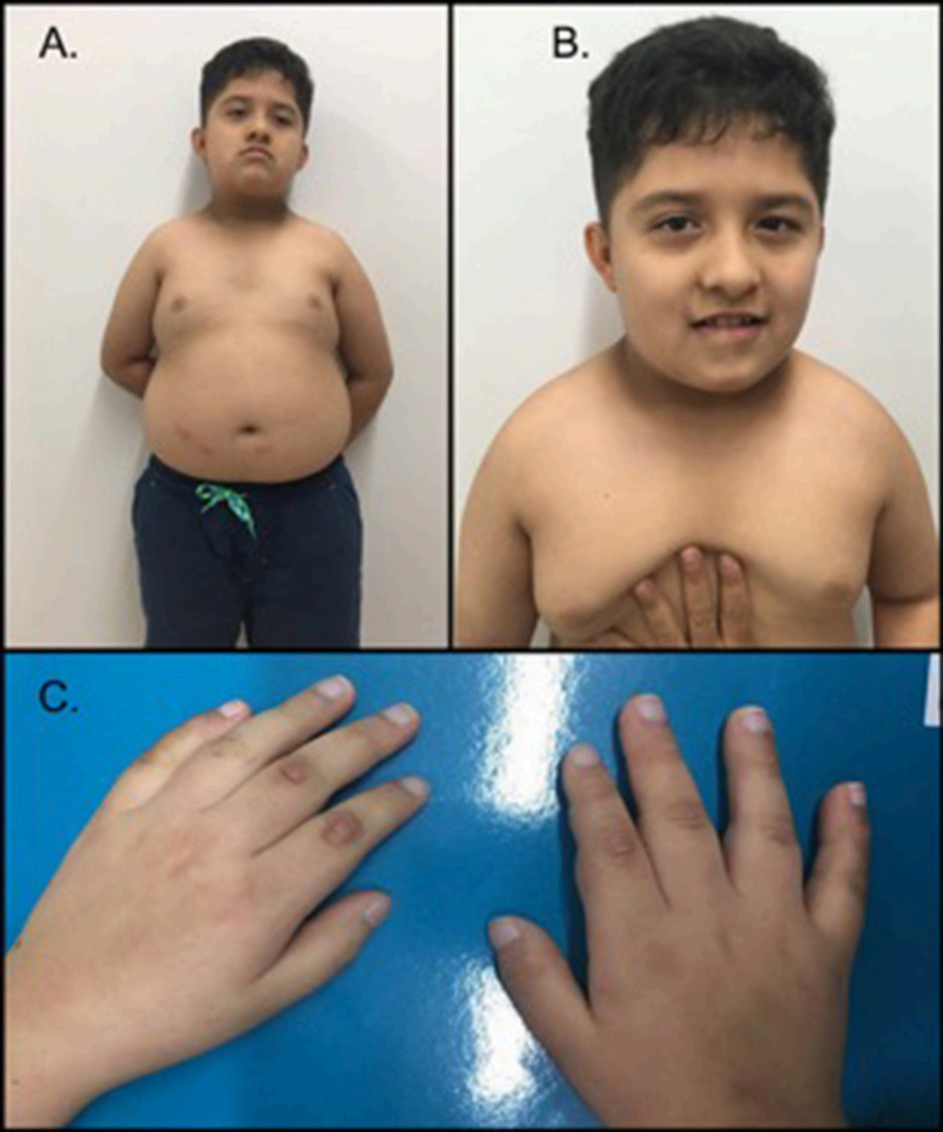
Phenotype of the patient. **(A)** The patient exhibited a short neck, brachycephaly, acanthosis nigricans on the nape of the neck, central abdominal obesity and gynecomastia. **(B)** Facial features demonstrated synophrys, facial asymmetry, downward slanting palpebral fissures, a broad nasal bridge and a thin upper lip. **(C)** Hands of the proband; note the short and thick fingers. The patient's parents provided written informed consent for the publication of his images.

On physical exam during a follow-up visit at age 10, the following findings were observed: weight = 44.4 kg (>87th perc.; z-score 1.16), height = 1.22 m (<1st perc.; z-score −3.0), acanthosis nigricans on the nape of the neck, brachycephaly, flat occiput, synophrys, small antihelix, facial asymmetry, distended abdomen, and a redundant prepuce (Figure 1). During this visit, the parents reported that the patient ate excessively and did not sleep for an adequate amount of time.

The patient continues to receive physical, speech, aquatic and occupational therapies and hippotherapy, most of which were initiated at 4 months of age. In line with the parents' wishes, the patient has not been enrolled in school or received formal education. Written informed consent was obtained from patient's parents for use of his images and clinical data for scientific purposes. Data were collected in the context of studies performed in accordance with the Declaration of Helsinki Good Clinical Guidelines and protocol #509 “registry of surveillance and survival congenital defects of the Colombian South-West” approved by Ethics Committee of Universidad Icesi (Act 192/2011).

## Discussion and conclusions

PWLS phenotype has been described in patients with 1p, 2p, 3p, 6q, 9q chromosome abnormalities, in cases with maternal uniparental disomy of chromosome 14 and fragile X syndrome (Gillessen-Kaesbach and Horsthemke, 1994; Eugster et al., 1997; Berends et al., 1999; Faivre, 2002; Cormier-Daire, 2003). A systematic review found that the most common clinical manifestations of PWLS include obesity (84%), hyperphagia (72.7%), mental disability (54.5%), psychomotor delay (50%), and hypotonia (43.18%; Rocha and Paiva, 2014).

Thirty-five case reports in the literature have linked the PWLS phenotype to a 6q16 deletions(Villa et al., 1995; Le Caignec et al., 2005; Varela et al., 2006; Klein et al., 2007; Bonaglia et al., 2008; Izumi et al., 2013), which is the most common genetic abnormality in patients exhibiting the PWLS phenotype. The majority of affected patients reported in the literature (22/35) were males, as in the present case. Many of these cases reviewed by El Khattabi et al. (2015) share the following features with the present case: Developmental delay (97%), learning disabilities (97%), obesity (65%), abnormal extremities (65%), behavioral problems (61%), vision anomalies (56%), rounded face/full cheeks (55%), and skull features (53%). Of the infrequently-reported features reported by Khattabi et al. including genital anomalies (11%), sleep disorders (16%), a bulbous nose (21%), and perinatal feeding difficulties (24%), only sleep disorders were observed in the present patient. Neonatal hypotony is seen in also a rare feature in 6q21 deletions that is observed in this case (Bonaglia et al., 2008). Other infrequently-reported features in the literature, including skin picking, high pain threshold, and decreased vomiting, were not observed in the present patient (Varela et al., 2006). Clinical features of previously reported patient with PWLS and deletions 6q are summarized in Table S1.

Here, we present a patient with the PWS phenotype but with no underlying mutation in the 15q11-q13 region, which would otherwise define classic PWS. In the absence of this mutation, the patient has been diagnosed with PWLS. The genetic etiology for this patient's condition is a 14.97 Mb deletion from 6q16.1-q21. 6q16 deletion is the most common genetic abnormality in patients exhibiting the PWS-like phenotype, our case is a 6q16.1 and 1q21 deletion, one case with novo balanced translocation between chromosomes 1p22.1 and 6q16.2 were published (Holder et al., 2000), however a concomitant deletion in both region has been reported in small amount of cases (Varela et al., 2006). These genotype is exceedingly rare, having been reported in the literature in nine other cases (Villa et al., 1995; Le Caignec et al., 2005; Varela et al., 2006; Klein et al., 2007; Bonaglia et al., 2008; Izumi et al., 2013).

Recently literature suggests that obesity and PWLS syndrome have are attributable to loss-of-function variants in the single-minded 1 (*SIM1*) gene included in the 6q16 critical minimal region (Holder et al., 2000; Bonnefond et al., 2013; Ramachandrappa et al., 2013). *SIM1* encodes a transcription factor that mediates hypothalamic paraventricular nucleus development. Loss-of-function variants in *SIM1* may cause human hyperphagic obesity with or without PWS-like features, and additionally may be responsible for neurobehavioral disorders (Bonnefond et al., 2013). Thus, haploinsufficiency of the SIM1 gene could be responsible for severe obesity in cases such as the present one. Additionally, it has been suggested that *GRIK2* deletions play a role in behavior disorders (Bonaglia et al., 2008). Observations have identified that the minimal critical region for PWLS is 1 Mb (Bonnefond et al., 2013). This region contains the *SIM1, MCHR2*, and *ASCC3* genes. In addition, *MCHR2* which encodes a receptor for melanin-concentrating hormones is known to increase food intake and body weight in mice. Therefore, as multiple genes with 6q16 region are important to control appetite and metabolism, a cumulative effect may cause the PW like phenotype (Spreiz et al., 2010; Rosenfeld et al., 2012; Ghoussaini et al., 2017).

Genomic imprinting or epigenetic factors might be mechanisms that mediate the variability of chromosome defects leading to PWLS. An imprinting effect in 6q16 deletions has been hypothesized based on the paternal origin of a *de novo* 6q16 deletion in a patient with PWLS (Faivre, 2002). It is possible that the phenotype is due to the haploinsufficiency of paternally expressed genes located in the deleted region (Spreiz et al., 2010). On the other hand, a few cases have been described with parental-origin deletion. To date, there is no strong evidence supporting an imprinting mechanism in the 6q16 region (Holder et al., 2000).

To the best of our knowledge, only one of the cases previously reported in the literature came from Latin American (Varela et al., 2006). All other reported cases are from North America (12 cases; Klein et al., 2007; Izumi et al., 2013) or Europe (22 cases; Villa et al., 1995; Faivre, 2002; Bonaglia et al., 2008). This patient is the first reported case from Colombia and the second reported case in Latin America, where one patient was reported previously for Varela et al. (2006).

It is challenging for clinicians to make a diagnosis when confronted with patients presenting with PWLS. If genetic analysis rules out PWS, an alternative diagnosis must be sought. Clinical suspicion of PWLS should be raised in the presence of features that have been commonly described in previous cases.

## Author contributions

All authors listed, have made substantial, direct and intellectual contribution to the work, and approved it for publication.

### Conflict of interest statement

The authors declare that the research was conducted in the absence of any commercial or financial relationships that could be construed as a potential conflict of interest.

## References

[B1] Berends M. J. W. Hordijk R. Scheffer H. Oosterwijk J. C. Halley D. J. Sorgedrager N. (1999). Two cases of maternal uniparental disomy 14 with a phenotype overlapping with the Prader-Willi phenotype. Am. J. Med. Genet. 84, 76–79. 10.1002/(SICI)1096-8628(19990507)84:1<76::AID-AJMG16>3.0.CO;2-F 10213052

[B2] Bonaglia M. C. Ciccone R. Gimelli G. Gimelli S. Marelli S. Verheij J.. (2008). Detailed phenotype-genotype study in five patients with chromosome 6q16 deletion: narrowing the critical region for Prader-Willi-like phenotype. Eur. J. Hum. Genet. 16, 1443–1449. 10.1038/ejhg.2008.119 18648397

[B3] Bonnefond A. Raimondo A. Stutzmann F. Ghoussaini M. Ramachandrappa S. Bersten D. C.. (2013). Loss-of-function mutations in SIM1 contribute to obesity and Prader-Willi–like features. J. Clin. Invest. 123, 3037–3041. 10.1172/JCI68035 23778136 PMC3696559

[B4] Cheon C. K. (2016). Genetics of Prader-Willi syndrome and Prader-Will-like syndrome. Ann. Pediatr. Endocrinol. Metab. 21, 126–135. 10.6065/apem.2016.21.3.126 27777904 PMC5073158

[B5] Cormier-Daire V. (2003). Cryptic terminal deletion of chromosome 9q34: a novel cause of syndromic obesity in childhood? J. Med. Genet. 40, 300–303. 10.1136/jmg.40.4.300 12676904 PMC1735435

[B6] El Khattabi L. Guimiot F. Pipiras E. Andrieux J. Baumann C. Bouquillon S.. (2015). Incomplete penetrance and phenotypic variability of 6q16 deletions including SIM1. Eur. J. Hum. Genet. 23, 1010–1018. 10.1038/ejhg.2014.230 25351778 PMC4795105

[B7] Eugster E. A. Berry S. A. Hirsch B. (1997). Mosaicism for deletion 1p36.33 in a patient with obesity and hyperphagia. Am. J. Med. Genet. 70, 409–412. 10.1002/(SICI)1096-8628(19970627)70:4<409::AID-AJMG14>3.0.CO;2-L 9182783

[B8] Faivre L. (2002). Deletion of the SIM1 gene (6q16.2) in a patient with a Prader-Willi-like phenotype. J. Med. Genet. 39, 594–596. 10.1136/jmg.39.8.594 12161602 PMC1735217

[B9] Ghoussaini M. Vatin V. Lecoeur C. Abkevich V. Younus A. Samson C.. (2017). Genetic study of the melanin-concentrating hormone receptor 2 in childhood and adulthood severe obesity. J. Clin. Endocrinol. Metab. 92, 4403–4409. 10.1210/jc.2006-2316 17698913

[B10] Gillessen-Kaesbach G. Horsthemke B. (1994). Clinical and molecular studies in fragile X patients with a Prader-Willi-like phenotype. J. Med. Genet. 31, 260–261. 10.1136/jmg.31.3.260-b 8014984 PMC1049764

[B11] Holder J. L. Jr. Butte N. F. Zinn A. R. (2000). Profound obesity associated with a balanced translocation that disrupts the SIM1 gene. Hum. Mol. Genet. 9, 101–108. 10.1093/hmg/9.1.101 10587584

[B12] Izumi K. Housam R. Kapadia C. Stallings V. A. Medne L. Shaikh T. H.. (2013). Endocrine phenotype of 6q16.1-q21 deletion involving SIM1 and Prader-Willi syndrome-like features. Am. J. Med. Genet. 161A, 3137–3143. 10.1002/ajmg.a.36149 24038875

[B13] Klein O. D. Cotter P. D. Moore M. W. Zanko A. Gilats M. Epstein C. J.. (2007). nInterstitial deletions of chromosome 6q: genotype-phenotype correlation utilizing array CGH. Clin. Genet. 71, 260–266. 10.1111/j.1399-0004.2007.00757.x 17309649

[B14] Le Caignec C. Swillen A. Van Asche E. Fryns J.-P. Vermeesch J. R. (2005). Interstitial 6q deletion: clinical and array CGH characterisation of a new patient. Eur. J. Med. Genet. 48, 339–345. 10.1016/j.ejmg.2005.04.010 16179229

[B15] Ramachandrappa S. Raimondo A. Cali A. M. Keogh J. M. Henning E. Saeed S.. (2013). Rare variants in single-minded 1 (SIM1) are associated with severe obesity. J. Clin. Invest. 123, 3042–3050. 10.1172/JCI68016 23778139 PMC3696558

[B16] Rocha C. F. Paiva C. L. A. (2014). Prader-Willi-like phenotypes: a systematic review of their chromosomal abnormalities. Genet. Mol. Res. 13, 2290–2298. 10.4238/2014.March.31.9 24737477

[B17] Rosenfeld J. A. Amrom D. Andermann E. Andermann F. Veilleux M. Curry C. (2012). Genotype-phenotype correlation in intersrtitial 6q deletion: a report of 12 new cases. Neurogenetics 13, 31–47. 10.1007/s10048-011-0306-5 22218741

[B18] Spreiz A. Müller D. Zotter S. Albrecht U. Baumann M. Fauth C.. (2010). Phenotypic variability of a deletion and duplication 6q16.1 → q21 due to a paternal balanced ins(7;6)(p15;q16.1q21). Am. J. Med. Genet. 152A, 2762–2767. 10.1002/ajmg.a.33699 20954245

[B19] Varela M. C. Simões-Sato A. Y. Kim C. A. Bertola D. R. De Castro C. I. Koiffmann C. P. (2006). A new case of interstitial 6q16.2 deletion in a patient with Prader–Willi-like phenotype and investigation of SIM1 gene deletion in 87 patients with syndromic obesity. Eur. J. Med. Genet. 49, 298–305. 10.1016/j.ejmg.2005.12.002 16829351

[B20] Villa A. Urioste M. Bofarull J. M. Martínez-Frías M. L. (1995). *De novo* interstitial deletion q16.2q21 on chromosome 6. Am. J. Med. Genet. 55, 379–383. 10.1002/ajmg.1320550326 7726240

